# Mid-Term Results of a Cemented Titanium–Niobium Nitride-Coated Mobile Knee in Primary Total Knee Arthroplasty

**DOI:** 10.3390/jcm14186357

**Published:** 2025-09-09

**Authors:** Serdar Jure, Mislav Čimić, Domagoj Delimar

**Affiliations:** 1 Department of Orthopaedic Surgery, University Hospital Center Zagreb, 10000 Zagreb, Croatia; mcimic@kbc-zagreb.hr (M.Č.); predstojnik.ort@kbc-zagreb.hr (D.D.); 2 Department of Orthopaedic Surgery, School of Medicine, University of Zagreb, 10000 Zagreb, Croatia

**Keywords:** total knee arthroplasty (TKA), titanium–niobium nitride (TiNbN), implant survivorship, patient-reported outcomes

## Abstract

**Objectives:** Materials with ceramic surface treatments have been adopted in total knee arthroplasty (TKA) to limit polyethylene wear and thus extend implant longevity. This study evaluated, at a single center, mid-term survivorship and clinical outcomes for a mobile-bearing knee prosthesis with a titanium–niobium nitride (TiNbN) coating. **Methods:** A total of 150 patients who underwent primary cemented TKA using the same TiNbN-coated mobile-bearing prosthesis were identified through the institutional database. Of these, 102 patients (102 knees) attended the follow-up examination and provided informed consent to participate in this study. All patients underwent comprehensive clinical and radiological assessment. Primary outcomes were the Forgotten Joint Score (FJS) and the Knee Injury and Osteoarthritis Outcome Score (KOOS). **Results:** After a mean follow-up period of 7.9 years, two revision surgeries were recorded. One revision was performed due to late periprosthetic joint infection, while the other involved synovectomy and liner exchange due to persistent stiffness and pain. At 9 years follow-up, overall survivorship of the TiNbN-coated TKA was 97.1% (95% CI, 88.4–99.3%). Mean FJS and KOOS were 70.0 (range 29–100) and 70.6 (range 24–98), respectively. No sex-based differences were detected in clinical outcomes or implant survivorship. **Conclusions:** The TiNbN-coated mobile-bearing knee prosthesis demonstrated favorable mid-term survivorship and patient-reported outcomes. These findings support its use as a treatment option for knee osteoarthritis, with performance comparable to contemporary TKA designs.

## 1. Introduction

The volume of primary and revision total knee arthroplasty (TKA) continues to rise in Europe and worldwide, particularly among younger patients, thereby increasing the need to address implant-related issues [1,2]. Moreover, registry data continue to show that increasing incidence of primary and revision TKA has a significant impact on healthcare costs and resource allocation [3]. Currently, it is expected that modern contemporary prosthetic implants might have a longer survivorship, as life expectancy is increasing while the patient mean age at the time of TKA is decreasing [1].

Because aseptic loosening is a leading cause of late failure after primary TKA [3], alternative bearing surface treatments, including ceramic coatings, have been developed to enhance the tribological performance of cobalt–chrome–molybdenum (CoCrMo) femoral components and thereby reduce polyethylene wear and loosening risk [4,5]. In parallel, there is growing interest in hypoallergenic implants, particularly in younger and more active patients, to address concerns pertaining to metal hypersensitivity and long-term wear performance [4].

Ceramic coatings are used in TKA not only to minimize wear for increasing implant longevity but also to reduce the allergenic potential of CoCrMo prosthetic components.

Ceramic coatings produced via nitrogen-based physical vapor deposition include titanium nitride (TiN), titanium–niobium nitride (TiNbN), and multilayer zirconium nitride (ZrN-CN-CrCN). When applied over the entire CoCrMo surface, these coatings function as a corrosion-resistant barrier, effectively rendering the implants “hypoallergenic” [4,5]. In vitro work has shown that such coatings can substantially lower metal-ion release from CoCrMo femoral components attributable to corrosion and wear [6]. Furthermore, systematic reviews published within the last three years confirm that ceramic-coated implants, while not demonstrating clear clinical superiority, remain a viable alternative to conventional cobalt–chrome designs, especially when metal-related reactions are suspected or high risk [7,8,9]. These recent findings highlight the clinical relevance of evaluating the medium- to long-term outcomes of TiNbN-coated prostheses as they may help refine implant selection strategies in contemporary TKA practice.

To date, several clinical series report mid-term clinical outcomes and survivorship for coated and uncoated TKAs to be broadly comparable in patients with and without a history of metal hypersensitivity, although longer-term data remain limited [4,10].

This single-center study reports mid-term survivorship and clinical outcomes for a consecutive series of TiNbN-coated mobile-bearing TKAs in patients without a documented history of metal allergy.

## 2. Materials and Methods

We conducted a retrospective, observational case series within a single-surgeon practice. Inclusion criteria comprised primary TKA for any indication, a minimum of 5-year follow-up, and use of the same TiNbN-coated prosthetic design. All patients who underwent TKA in the same orthopedic center between October 2015 and December 2016 and who met these criteria were identified as eligible. Exclusion criteria included revision TKA, prior ipsilateral knee surgery, inflammatory arthritis, and incomplete clinical data. All patients followed a standardized postoperative rehabilitation protocol consisting of early mobilization, supervised physiotherapy, and progressive weight-bearing. The potential effect of BMI on outcomes was not analyzed in this case series.

This study was approved by the local ethics committee and was conducted in accordance with the ethical standards laid down by the 1964 Declaration of Helsinki and its later amendments. All patients enrolled gave their written informed consent. The primary study end-point was defined as the cumulative probability of implant survival. Secondary end-points were any other complications, reoperations, and clinical outcomes.

### 2.1. Implant Description

**Figure 1:**
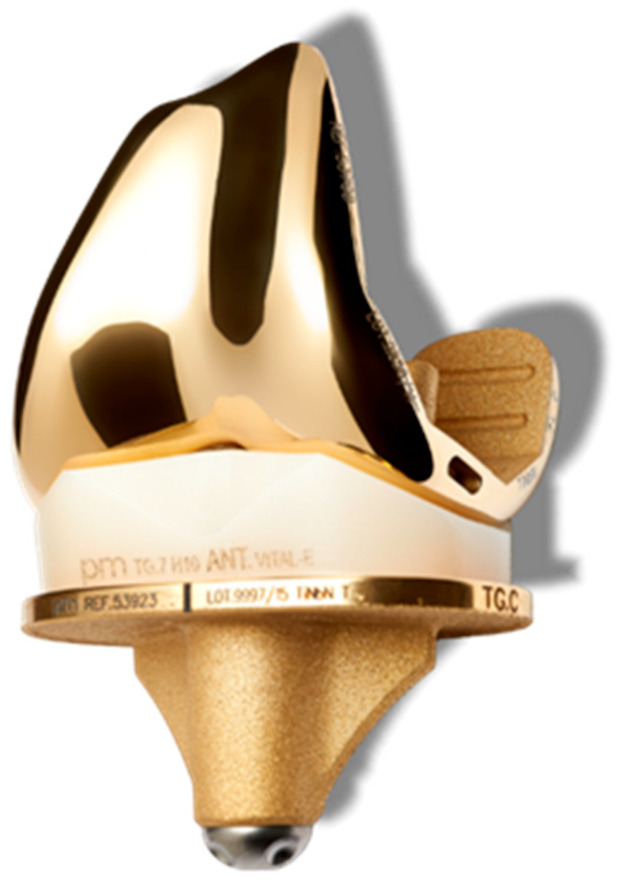
Picture of the cemented rotating-platform TiNbN-coated knee prosthesis, GKS Prime Flex Mobile Bioloy^®^ by Permedica Orthopaedics, Merate, Italy. Image courtesy of Permedica Orthopaedics.

The investigated prosthetic implant was a cemented rotating-platform bicompartmental knee prosthesis (GKS Prime Flex Mobile Bioloy^®^, Permedica Orthopaedics, Merate, Italy). The femoral component was a J-curve design made with CoCrMo. The tibial component was made by a forged titanium alloy. Both components were coated with a TiNbN monolayer (Bioloy^®^, Merate, Italy). All knees included in this study received the same knee prosthesis with an ultra-congruent rotating insert in vitamin E-blended polyethylene or conventional polyethylene (Figure 1).

### 2.2. Surgical Procedure

A single surgical team led by the senior surgeon (D.D.) performed all procedures. Spinal anesthesia was the default technique, whereas general anesthesia was used selectively for spinal deformities. Tranexamic acid was intravenously administered before surgery and topically applied when needed. All operations were carried out under tourniquet control. Exposure was obtained through a straight anterior midline incision and a medial parapatellar arthrotomy. Mechanical alignment guided both femoral and tibial resections. Radiopaque, antibiotic-free bone cement was used to fix all components beneath the tibial plateau and femoral condyles. All cases included patellar resurfacing. Use of the hypoallergenic implant did not depend on documented or suspected cutaneous metal allergy.

### 2.3. Study Procedure

A search on the hospital database was carried out to identify eligible patients, using references to the investigated implant as search terms. All medical records were selected from the hospital database and reviewed for those patients who met all inclusion criteria. Patient contact data, age at operation, gender, BMI, diagnosis, date of intervention, surgical procedure, and any following reintervention or revision surgery were registered.

A total of 150 patients (150 knees) were identified as eligible. Out of these, 25 patients died for causes not related to their implants, and 23 patients were lost to follow-up, leaving 102 patients (102 knees) available for the study.

Eligible patients were approached by telephone. We obtained informed consent and recorded the current clinical status of the patients, together with any postoperative complications or reoperations.

### 2.4. Clinical Assessment

At the latest review, clinical status was captured using two patient-reported outcome measures (PROMs). Patients completed the Forgotten Joint Score (FJS-12) questionnaire [11]. Patients were asked to answer all 12 question about their current quality of life and the conditions of their operated knee. They also completed the Knee Injury and Osteoarthritis Outcome Score (KOOS) questionnaire at follow-up [12].

### 2.5. Failures and Complications

We recorded all intra- and postoperative complications, as well as any revisions and reinterventions, for all TKAs. Revision was defined as removal and replacement of one or more prosthetic components. Reintervention was defined as a knee reoperation in which no prosthetic components were removed.

### 2.6. Statistical Analysis

We estimated implant survivorship with the Kaplan–Meier method using endpoints of revision for any cause and revision for aseptic loosening. The associated 95% confidence intervals are reported. Categorical variables were compared using χ^2^ tests or Fisher’s exact test, as appropriate. For continuous data, we used Student’s *t*-test when normality was satisfied; otherwise, we used the Mann–Whitney U test. Distribution normality was evaluated with the Shapiro–Wilk and D’Agostino–Pearson tests. Two-sided *p*-values ≤ 0.05 were deemed statistically significant. All analyses were performed in GraphPad Prism v10.1.0 (Boston, MA, USA).

## 3. Results

Among the 102 knees analyzed, the mean age at surgery was 64 years (range: 35–80), and 78 patients (76.5%) were female. Mean BMI was 27 (range: 24–31). Indication in all knees was end-stage osteoarthritis. Five women had a known history of nickel hypersensitivity. The remaining patients had no prior metal allergy history. Mean follow-up was 7.9 years (range: 1.0–9.2). At latest follow-up, mean FJS was 70.0 (range: 26–100) and mean KOOS was 70.6 (range: 24–98). A summary of KOOS domain scores is provided in Table 1. The lowest scores occurred in the Sport domain, reflecting greater difficulty with squatting, running, jumping, and kneeling. Except for those who required additional reoperation, patients reported being satisfied with the implant.

There were no major intraoperative complications. A patient experiencing pain and stiffness at 1 year underwent synovectomy with liner exchange. Intraoperative microbiology from this case excluded periprosthetic infection. In addition, no macroscopic signs of particle disease or aseptic loosening were seen intraoperatively.

**Figure 2:**
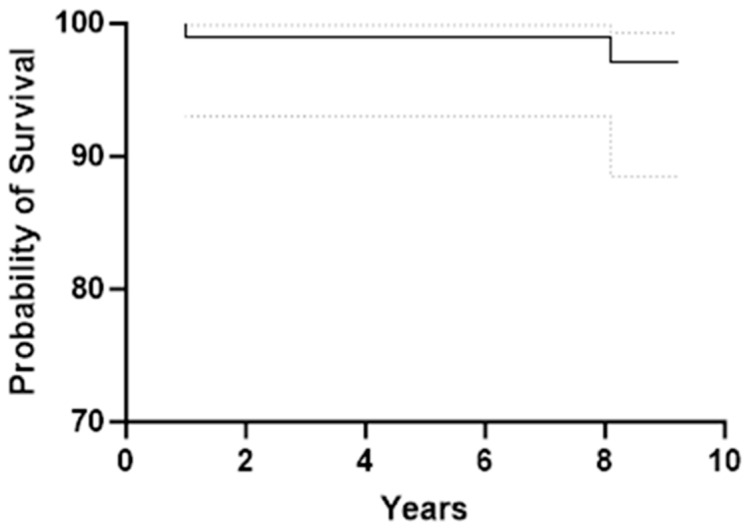
Kaplan–Meier survival curve for TiNbN-coated mobile-bearing TKA considering revision for any reason as endpoints. The solid line represents the cumulative implant survival probability, while the dotted lines indicate the 95% confidence interval. The overall survival rate was 97.1% at 9 years (95% CI: 88.4–99.3%).

A further late deep periprosthetic infection required full component exchange 8 years after the index procedure. No cases underwent revision for aseptic loosening. Nine-year survivorship was 97.1% (95% CI, 88.4–99.3%) for any cause and 100% for aseptic loosening (Figure 2). There were no statistically significant sex-based differences in survivorship or PROMs; however, women showed numerically lower values (Table 2).

## 4. Discussion

Traditionally, TKA femoral components are made from CoCrMo alloy. Alternative materials comprise oxidized zirconium (Oxinium™), titanium alloys, alumina-reinforced matrices, and, more recently, reinforced PEEK.

In addition to standard CoCrMo, numerous coatings have been applied to femoral bearing surfaces and mobile tibial components. These components are commonly described as “alternate-surface,” “ceramic-coated,” or “ceramicised.” Ceramic coatings used in clinical practice include zirconium nitride multilayer coating and titanium nitride and titanium–niobium nitride coatings.

In patients with known or suspected cutaneous metal allergy, ceramic-coated implants are often chosen because they can mitigate metal-ion release from CoCrMo substrates. These ceramic-coated CoCrMo implants are in fact also referred to as “hypoallergenic” implants [4,10].

The findings of the current study support the use of hypoallergenic ceramic-coated knee implants in primary TKA in patients with unknown or no previous history of metal allergy. Our survivorship and PROMs are consistent with reports for comparable devices at similar follow-up intervals [10,11,12]. No significant differences were observed between male and female patients. Our data indicate that TiNbN-coated knees perform on par with uncoated prostheses, in line with current evidence; thus, TiNbN-coated devices may be a reasonable option when the goal is to reduce the risk of rare metal-related reactions in patients without known dermal allergies. In addition, particle-induced synovitis after TKA has been reported as a potential complication, arising from prosthesis wear debris and metal particle release [13]. Although we did not directly evaluate this in our cohort, it remains an important consideration for long-term implant performance.

Original articles on clinical studies with ceramic-coated knee prostheses almost agree reporting comparable survival, radiological, and clinical outcomes at mid-term follow-up between ceramic-coated and uncoated TKA [8]. So far, clinical studies have not shown ceramic-coated implants to be superior to uncoated CoCrMo in primary TKA regarding outcomes, survivorship, or revisions for wear-related aseptic loosening up to 10 years [14,15,16,17,18].

Randomized trials, to date, have likewise not demonstrated clinical superiority, changes in inflammatory markers, or reduced systemic metal-ion levels for coated versus standard implants [19,20,21,22].

One of these ceramic-coated knee systems which claims longer follow-ups is the TiN-coated ACS knee (Implantcast GmbH, Buxtehude, Germany). Several authors from different countries have reported excellent survivorships for large consecutive series with the ACS mobile bearing knee in the mid and long term, even up to 16-year follow-up, with an acceptable revision rate [7,13,23].

Analyses from the German Arthroplasty Registry (EPRD) do not show a survivorship advantage for ceramic-coated components, including regarding revisions for periprosthetic joint infection [24].

The Australian registry has reported higher revision rates for alternate-surface femoral components compared with conventional CoCr components [3]. In particular, the revision rate is 5.8% (95% CI: 5.1–6.6%) at 10 year follow-up when TiN femoral component is used against 4.4% (95% CI: 4.4–4.5%) with other uncoated femoral components [3]. Interestingly, the revision rate for aseptic loosening and for pain or patellofemoral pain is increased when alternate surface femoral components have been used in comparison with uncoated femoral components [3]. This finding could be related to the suboptimal tribological properties of such ceramic coatings, or, otherwise, to patients’ related characteristics as higher BMI can be found in the Australian population than in the European population, but these hypotheses have not yet been proven.

Taken together, the literature indicates that hypoallergenic knees exhibit performance and failure profiles comparable to standard uncoated designs, including at longer follow-up. So, the use of such hypoallergenic implants—and, consequently, their increased price over standard implants—might be justified considering that potential adverse immunological reactions to CoCrMo implants could be prevented [25].

In hypoallergenic designs, a ceramic coating provides an inert, biocompatible barrier across the whole surface, limiting direct CoCrMo contact with periarticular tissues and reducing ion release from the metallic substrate. This isolating barrier might thus delay or stop the triggering of possible metal hypersensitivity reactions to one or more metal elements. These metal hypersensitivity reactions, sometimes reported as metal allergies or metal adverse biologic reactions, although very rare, always lead to an early painful knee, patient dissatisfaction, functional impairment, and early implant failure [5,26]. The prevalence of TKA failure due to metal hypersensitivity is uncertain and likely underestimated because of diagnostic challenges [5,26]. Importantly, cutaneous patch-test positivity reflects a different biological mechanism than delayed type-IV hypersensitivity to implanted metals. Different cells and different biologic mechanisms are involved, and these two different mechanisms seem to be not correlated [27,28]. Although many studies report the prevalence of skin metal allergies, few quantify the revisions that are attributable to metal hypersensitivity, which may be <0.05% after TJA [29]. The Australian Joint Replacement Registry reported the reason for the revision of 104 primary TKAs for osteoarthritis as a “metal-related pathology,” with a prevalence of 0.01%, and the reason for the revision of 1995 primary TKAs was reported as “pain” [3]. It is reasonable to assume that among the knees undergoing revision for unexplained pain, some of these could be due to unrecognized metal hypersensitivity. However, our study did not include systematic allergy testing before or after surgery, which represents a limitation in interpreting whether TiNbN coating benefits patients with true metal hypersensitivity.

Despite limited high-level evidence in primary TKA, recent reports suggest zirconium–nitride multilayer coatings may be useful in revision TKA for suspected metal hypersensitivity after primary uncoated implants [25,30]. One limitation of this study is the lack of preoperative FJS and KOOS data, which prevents a direct assessment of functional improvement after surgery.

Some concerns still remain unsolved regarding hypoallergenic knee prostheses and the wear behavior of ceramic coatings against polyethylene liners over time. Retrieval studies show that despite good adhesion and surface integrity, these coatings undergo in vivo wear and degradation with heterogeneous damage patterns [31,32,33]. Cost is an important consideration; because hypoallergenic implants carry a price premium, cost-effectiveness should be weighed carefully in the absence of proven advantages in survivorship or function. Given the absence of a control cohort, a formal economic evaluation was beyond the scope of this study.

This study has several limitations. First, the absence of a control group with uncoated implants limits direct comparisons of outcomes and survivorship. Second, the modest cohort size and single-center design reduce generalizability, while loss to follow-up may have introduced selection bias. Third, preoperative baseline PROMs were not collected, preventing the assessment of improvement magnitude after surgery. Fourth, allergy testing was not systematically performed, which restricts conclusions regarding the specific benefit of TiNbN coating in patients with true metal hypersensitivity. Finally, we did not undertake a formal cost-effectiveness analysis and did not capture device-level pricing; accordingly, we cannot comment on the economic justification for the routine use of coated implants beyond selected scenarios. Moreover, because only a single postoperative KOOS was available, comparisons with other series are descriptive; future work should incorporate pre–post change scores and responder analyses to facilitate benchmarking.

To overcome these limitations, future research should focus on larger multicenter randomized controlled trials with longer follow-up periods to determine whether ceramic-coated implants offer measurable clinical or economic advantages over conventional designs. Retrieval studies of TiNbN-coated implants are also warranted to better understand the long-term wear mechanisms and coating durability. Moreover, incorporating systematic allergy testing could clarify the role of ceramic coatings in preventing adverse biological reactions, thus guiding personalized implant selection in primary and revision TKA.

## 5. Conclusions

In summary, a cemented TiNbN-coated mobile-bearing knee prosthesis demonstrated satisfactory mid-term survivorship and favorable patient-reported outcomes, with results comparable to uncoated designs. The innovative aspect of this study lies in its providing mid-term clinical evidence for TiNbN-coated implants in patients without prior metal hypersensitivity, supporting their use as a safe and effective alternative in primary TKA. While our data align with recent registry and clinical reports, they also emphasize that the main potential benefit of ceramic coatings may not be in superior survivorship but in offering an additional layer of safety against rare but clinically significant metal-related reactions.

## Figures and Tables

**Table 1:** Mean scores, with ranges, of the Knee Injury and Osteoarthritis Outcome Score (KOOS)—five dimensions.

KOOS Section	Mean	Range
Symptoms	75.7	36–100
Pain	86.6	36–100
Function of daily living	83.0	62–100
Sport	37.8	0–100
Quality of life	70.5	0–100

**Table 2:** Comparison of demographic and clinical outcomes between male and female patients who underwent TiNbN-coated mobile-bearing total knee arthroplasty.

Parameter	Male Group	Female Group	*p*-Value
Number	24	78	-
Mean age (SD, range)	62.2 (8.1, 36–75)	64.6 (8.1, 35–80)	0.2426
Mean BMI (SD, range)	26 (1.1, 25–31)	25 (1.3, 24–30)	0.3562
Mean follow-up	7.5	7.9	0.1626
Number of reinterventions	0	2	1.0000
Number of reinterventions without implant removal	0	1	1.0000
Number of revisions	0	1	1.0000
Survival rate (CI, 95%)	100%	96.2%	0.4702
Mean FJS (SD)	77.5 (19.8)	67.7 (21.4)	0.0596
Mean KOOS	76.3	68.9	0.0568

## Data Availability

The data presented in this study are available on request from the corresponding author.

## Abbreviations

TKA	Total Knee Arthroplasty
TiNbN	Titanium–Niobium Nitride
FJS	Forgotten Joint Score
KOOS	Knee Injury and Osteoarthritis Outcome Score
CoCrMo	Cobalt–Chrome–Molybdenum
TiN	Titanium Nitride
ZrN-CN-CrCN	Zirconium Nitride multilayer coating
PEEK	Polyetheretherketone
PROMs	Patient-Reported Outcome Measures
BMI	Body Mass Index
CI	Confidence Interval
